# Correlation between mutations and mRNA expression of *APC* and *MUTYH* genes: new insight into hereditary colorectal polyposis predisposition

**DOI:** 10.1186/s13046-015-0244-4

**Published:** 2015-10-28

**Authors:** Gitana Maria Aceto, Fabiana Fantini, Sabrina De Iure, Marta Di Nicola, Giandomenico Palka, Rosa Valanzano, Patrizia Di Gregorio, Vittoria Stigliano, Maurizio Genuardi, Pasquale Battista, Alessandro Cama, Maria Cristina Curia

**Affiliations:** Department of Medical, Oral and Biotechnological Sciences, “G. d’Annunzio” University, Chieti, Italy; Department of Clinical Physiopathology, University of Florence, Florence, Italy; Immunohaematology and Transfusional Medicine Service, “SS. Annunziata” Hospital, Chieti, Italy; Division of Gastroenterology and Digestive Endoscopy, Regina Elena National Cancer Institute, Rome, Italy; Institute of Medical Genetics, “A. Gemelli” School of Medicine, Catholic University of the Sacred Heart, Rome, Italy; Department of Pharmacy, “G. d’Annunzio” University, Chieti, Italy

**Keywords:** *APC*, *MUTYH*, Mutation, Adenomatous polyposis, Colon cancer, Phenotype, Gene expression, qRT-PCR, ASE, Predisposition

## Abstract

**Background:**

Transcript dosage imbalance may influence the transcriptome. To gain insight into the role of altered gene expression in hereditary colorectal polyposis predisposition, in the present study we analyzed absolute and allele-specific expression (ASE) of *adenomatous polyposis coli* (*APC*) and *mutY Homolog* (*MUTYH*) genes.

**Methods:**

We analyzed DNA and RNA extracted from peripheral blood mononuclear cells (PBMC) of 49 familial polyposis patients and 42 healthy blood donors selected according similar gender and age. Patients were studied for germline alterations in both genes using dHPLC, MLPA and automated sequencing. *APC* and *MUTYH* mRNA expression levels were investigated by quantitative Real-Time PCR (qRT-PCR) analysis using TaqMan assay and by ASE assays using dHPLC-based primer extension.

**Results:**

Twenty out of 49 patients showed germline mutations: 14 in *APC* gene and six in *MUTYH* gene. Twenty-nine patients did not show mutations in both genes. Results from qRT-PCR indicated that gene expression of both *APC* and *MUTYH* was reduced in patients analyzed. In particular, a significant reduction in *APC* expression was observed in patients without *APC* germline mutation *vs* control group (*P* < 0.05) while *APC* expression in the mutation carrier patients, although lower compared to control individuals, did not show statistical significance. On the other hand a significant reduced *MUTYH* expression was detected in patients with *MUTYH* mutations *vs* control group (*P* < 0.05). Altered ASE of *APC* was detected in four out of eight *APC* mutation carriers. In particular one case showed a complete loss of one allele. Among *APC* mutation negative cases, 4 out of 13 showed a moderate ASE. ASE of *MUTYH* did not show any altered expression in the cases analyzed. Spearman’s Rho Test analysis showed a positive and significant correlation between *APC* and *MUTYH* genes both in cases and in controls (*P* = 0.020 and *P* < 0.001).

**Conclusions:**

*APC* and *MUTYH* showed a reduced germline expression, not always corresponding to gene mutation. Expression of *APC* is decreased in mutation negative cases and this appears to be a promising indicator of FAP predisposition, while for *MUTYH* gene, mutation is associated to reduced mRNA expression. This study could improve the predictive genetic diagnosis of at-risk individuals belonging to families with reduced mRNA expression regardless of presence of mutation.

**Electronic supplementary material:**

The online version of this article (doi:10.1186/s13046-015-0244-4) contains supplementary material, which is available to authorized users.

## Introduction

Colorectal cancer (CRC) is one of the most frequent causes of cancer death worldwide [1, 2]. Inherited forms of CRC account for as much as 20–30 % of all CRC cases whereas hereditary colorectal polyposis syndromes account for about 1 % of all cases of CRC [3]. The identification of mechanisms predisposing to hereditary colorectal polyposis is necessary for direct genetic diagnosis of carrier status in cases without detectable mutations in known genes. Mendelian predisposition syndromes, like familial adenomatous polyposis (FAP), associated with mutations in known genes account for 5–10 % of the overall incidence of the disease [4, 5]. The genes mostly implicated in the inheritance of adenomatous polyposis, a condition that leads to colorectal cancer, are *adenomatous polyposis coli* (*APC*) involved in FAP (OMIM #175100) and *mutY Homolog* (*MUTYH*) involved in *MUTYH*-associated polyposis (MAP) (OMIM#608456). The tumor suppressor *APC* gene, a component of Wnt pathway, encodes a multifunctional protein that regulates many cellular processes, as differentiation, proliferation, life and death decision; it is involved in ephithelial turn-over, then APC loss is an early event in gut epithelium tumorigenesis [6]. *APC* germline mutations are detected in the majority of FAP patients, even if in a relevant subset of cases the mutations cannot be identified. A fraction (7–23 %) of *APC* mutation negative cases with phenotypes overlapping with attenuated FAP (AFAP) or classical FAP, is associated with biallelic germline variants of the *MUTYH* gene. *MUTYH* together with *OGG1* and *MTH1* is a component of DNA Base Excision Repair (BER) pathway that removes damaged bases generated by reactive oxygen species (ROS), capable to induce mutations commonly observed in cancerogenesis [7–9]. A relevant phenotypic pre-cancerous variability has been observed in kindred carrying the same germline mutation and literature data suggest that gene expression dosage can play a role as a new genetic mechanism for colorectal cancer susceptibility [10, 11]. The variation in gene expression contributes to phenotypic variability, plays an important role in the etiology of diseases and may affect absolute and allele specific expression [12–14]. In up to 50 % of polyposis families no germline mutation is identified and about 10–15 % of FAP patients could have a reduced *APC* expression with similar phenotype to patients with truncating *APC* mutation [15, 16]. Germline altered allele specific expression (ASE) is an indicator of genetic imbalances and a useful marker of predisposition to polyposis and/or colorectal cancer [10, 11, 17–19]. The dosage of the *APC* transcripts may modulate the disease resulting in classic or attenuated phenotype in cases with and without pathogenic mutations [17, 18, 20–22]. To gain insight in the correlation between *APC* and *MUTYH* mutations and altered expression, in the present study we investigated the role of dosage imbalance influencing the transcriptome of these two colon cancer-predisposing genes, performing an analysis of absolute and allele-specific expression in patients with different degrees of penetrance of hereditary colorectal disease. We analyzed peripheral blood mononuclear cells (PBMCs) from patients with and without mutations and compared the gene expression with control individuals. Finally, to understand the interaction between *APC* and BER pathway, we investigated the possible mutual modulation. This exploratory study on correlation among mutational spectrum, gene dosage and phenotype, could improve the genetic diagnosis performing predictive testing of at-risk individuals belonging to families with reduced mRNA expression regardless of presence of mutation. The identification of carriers of mutation or/and reduced mRNA expression may be helpful for the appropriate monitoring and clinical management of patients.

## Patients and methods

### Patients and nucleic acid preparation

We analyzed 49 polyposis patients recruited in different collaborating Italian Institutions including the Units of Clinical Physiopathology and Medical Genetics of the University of Florence, Medical Genetics of the University “G. d’Annunzio” of Chieti-Pescara, Gastroenterology and Digestive Endoscopy of the “Regina Elena” National Cancer Institute of Rome. This series consisted of patients affected by classical FAP, AFAP, multiple colorectal polyposis (with more than five polyps) [23] and familial colon cancer. Sixteen cases represented a research-based cohort from Aceto et al. [24]. Patients were selected based on availability of RNA and on the presence of at least 2–5 polyps at diagnosis in the probands and/or their relatives. One hundred and fifty healthy blood donors who reported no personal or family history of adenomas or colorectal cancer have been employed to identify and filter transcripts that exhibit altered transcript expression in the unaffected population. All study participants gave written informed consent after verbal counseling and the study was approved by the Ethics Committee of the University “G.d’Annunzio” of Chieti. Nucleic acids extraction from PBMCs and synthesis of complementary DNA (cDNA) from 3.5 μg of total RNA were performed as previously described [24]. Samples with poor quality or insufficient quantity of target in the cDNA template were not included in the mRNA expression analyses.

### Screening for sequence variants

Mutation screening of *APC* and *MUTYH* genes for patients analyzed in previous study was conducted with different PCR-based techniques as previously described [24]. Additional screening was conducted in this study using denaturing high performance liquid chromatography (dHPLC) and automated sequencing. For *APC* also multiplex ligation-dependent probe amplification (MLPA) analysis was performed using the SALSA P043 APC MLPA kit (MCR-Holland, Amsterdam, The Netherlands) to detect large gene-rearrangements. Mutations are listed in the International Society for Gastrointestinal Hereditary Tumours (InSiGHT, http://www.insight-group.org/variants/database/) database.

### Real-time quantitative PCR analysis (qRT-PCR)

The level of *APC* and *MUTYH* messenger RNA (mRNA) expression in PBMCs was investigated by TaqMan quantitative real-time PCR (qRT-PCR) analysis using StepOne™ 2.0 (Applied Biosystems). Data were analyzed using the comparative Ct method and were graphically indicated as 2^−^∆Ct ± SE. In accordance with the method, the mRNA amounts of the target genes (*APC* and *MUTYH* #Hs01568269_m1, #Hs01014856_m1 respectively, Applied Biosystems) were normalized to the endogenous housekeeping gene GUSB (#Hs99999908_m1, Applied Biosystems) [25]. Target and reference genes were amplified separately in triplicate for both cases and controls in a volume of 10 μl containing 1 μl template cDNA diluted 1:10, 0.5 μl of primers and probes mixture (20X FAM-labeled Assay-on-Demand Gene Expression Assay Mix) and 5 μl of 2X TaqMan Universal Master Mix (Applied Biosystems). The cycling conditions were performed as follows: 2 min at 50 °C, 10 min at 95 °C and 40 cycles of 15 s at 95 °C followed by 1 min at 60 °C.

### Allele-specific expression (ASE) analysis

ASE analyses were performed by dHPLC-based single nucleotide primer extension (SNuPE) [26]. This technique allows to measure the relative allele expression comparing the heights of the peaks corresponding to the two alleles obtained by PCR-amplified gDNA and cDNA templates. Overall mean ratio obtained by analyzing gDNA templates for the corresponding variant was used to normalize cDNAs. These normalized cDNA/gDNA ratios were designed as ASE values. ASE measures were performed multiple times and coefficient of variation (CV) was calculated to test for assay reproducibility. Single-nucleotide polymorphism (SNP) genotyping analysis was performed to detect allelic markers of heterozygosity useful to ASE analysis. The frequent and common SNPs rs2229992 (c.1458C > T) (minor allele frequency, MAF = 0.49) in exon 12 of the *APC* gene and rs3219489 (c.1014G > C) (MAF = 0.31) in exon 12 of *MUTYH* gene were selected. An additional specific assay on exon 7 frequent pathogenic mutation of *MUTYH* gene (rs34612342, c.536A > G) was also designed (Additional file 1: Table S1).

### Statistical analysis

The one-way analysis of variance (ANOVA) in the absolute expression of mRNA levels was performed to test the difference in means between different groups. Bonferroni post-hoc test allowed us to identify which group resulted significantly different based on the comparison of multiple values simultaneously.

ASE values were summarized as median, mean and standard deviation (SD) separately for the two groups (cases and controls). Shapiro-Wilk test was used to evaluate the normality in the distribution within each group. To assess differences of ASE values between cases and controls the Kruskall-Wallis test was performed followed by other tests carried out to determine if different proportions of individuals in cases and controls group were at a standard distance from the overall mean (1.0 SD, about 68 % of the distribution) by Chi-square test or Fisher’s exact test. To assess a possible correlation between the two genes Spearman’s rho correlation coefficient was evaluated. All analyses were performed with STATA software (version 10).

## Results

Cases were analyzed for *APC* and *MUTYH* germline mutations, absolute expression and ASE. All analyses were performed and compared to a control population.

### Screening for *APC* and *MUTYH* germline mutations

Mutation analysis detected pathogenic mutations in 20 patients out of 49 analyzed (Table 1). For *APC* gene 14 mutations introducing frameshift were detected, of which 12 previously published [2] and 2 detected in the present study, including a deletion of entire exon 15 and the p. Leu878_CysfsX916. Among these mutations only one (the p.His393_PhefsX396) fell in the gene portion associated to an attenuated phenotype [8, 21, 27]. This mutation was found in case GD37, and was located in the alternative splicing region of exon 10. Mutation analysis of *MUTYH* gene was performed for cases negative at *APC* mutation screening and detected sequence variants in six patients: two carriers of heterozygous variants (GD72 and GD155), three compound heterozygotes (GD68, GD82#1, GD82#2), and one carrier of exon 12 homozygous deletion (GD91) (Table 1). Four mutations were detected in a previous study [24] and 2 in the present study. Twenty-nine out of 49 patients did not show mutations in both genes (Table 2). Basic clinical features are reported in Tables 1 and 2; as noted polyposis patients without *APC* mutations manifested similar phenotype to patients with truncating *APC* mutations.

**Table 1:** Clinical and molecular characteristics of patients with mutations

Patients with *APC* mutations							
Case^a^	Age at diagnosis	Phenotype	Family History (transmission)	Additional informations	*APC* Mutation	Exon	Effect
GD22	45	FAP	yes (vertical)	osteomas, desmoids, duodenal cancer	c.646-1 G > A^b^	7	Criptic splice site fsX292
GD23	n.a.	FAP	yes		c.4666dup^b^	16	p.Thr1556_AsnfsX1558
GD31	72	FAP	yes (vertical)	>1000 polyps, colon cancer	c.3183_3187del^b^	16	p.Lys1061_LysfsX1062
GD33	n.a	FAP	yes (vertical)		c.904 C > T^b^	9	p.Arg302X
GD37	41	AFAP	yes (vertical)	ileal polyps, colon cancer,	c.1176_1177insT^b^	10	p.His393_PhefsX396
GD38	35	FAP	yes (vertical)	CHRPE, osteomas	c.4717 G > T^b^	16	p.Glu1573X
GD41	24	FAP	yes (vertical)	gastric and ileal polyps	c.2684 C > A^b^	16	p.Ser895X
GD48	33	FAP	yes (vertical)	duodenal polyps	c.2758_2759del^b^	16	p.Asp920_CysfsX922
GD57	22	FAP	no		c.2299 C > T^b^	16	p.Gln767X
GD58	48	FAP	no		c.4393_4394del^b^	16	p.Ser1465_TrpfsX1467
GD59	31	FAP	yes (vertical)		c.4192_4193del^b^	16	p.Ser1398_SerfsX1407
GD74	27	FAP	yes (vertical)	>100 polyps, desmoids, colon cancer	ex15del	15	∆ 15
GD103	55	FAP	no		c.694 C > T^b^	7	p.Arg232X
GD119	26	FAP	yes (vertical)	duodenal polyps, rectal cancer	c.2633delT	16	p.Leu878_CysfsX916
ᅟ	ᅟ	ᅟ	ᅟ	ᅟ	ᅟ	ᅟ	ᅟ
Patients with *MUTYH* mutations							
Case^a^	Age at diagnosis	Phenotype	Family History (transmission)	Additional informations	*MUTYH* Mutation	Exon	Effect
GD68	45	AFAP	no	40 polyps	[c.536A > G]+	7, 12	[p.Tyr179Cys]+
					[c.1163 T > C]^b^		[p.Leu388Pro]
GD72	29	FAP	yes (vertical)		[c.536A > G] + [c.=]^b^	7	[p.Tyr179Cys] + [p.=]
GD82#1	49	Multiple polyposis	yes (horizontal)	caecum cancer	[c.536A > G]+	7, 10	[p.Tyr179Cys]+
					[c.820C > T]^b^		[p.Arg274Trp]
GD82#2	54	Colon cancer	yes (horizontal)		[c.536A > G]+	7, 10	[p.Tyr179Cys]+
					[c.820C > T]		[p.Arg274Trp]
GD91	30	Multiple polyposis	no	100 polyps	[c.1145del]+	12	[p.Ala382AlafsX407]+
					[c.1145del]^b^		
							[p.Ala382AlafsX407]
GD155	70	AFAP	no		[c.536A > G] + [c.=]	7	[p.Tyr179Cys] + [p.=]

^a^Identification number is followed by number of individual if several members were investigated *per* family

^b^Data from [24]

*n.a.* = not available

**Table 2:** Clinical and molecular characteristics of patients without mutations

Patients without *APC* and *MUTYH* mutations				
Case^a^	Age at diagnosis	Phenotype	Family History (transmission)	Additional informations
GD70	43	AFAP	yes (vertical)	
GD78	35	Multiple polyposis	yes (vertical)	>10 polyps
GD80	45	Multiple polyposis	yes (horizontal)	
GD81	36	AFAP	yes (vertical)	
GD83	36	FAP	yes (horizontal)	
GD84	32	FAP	no	desmoids
GD86	17	FAP	yes (vertical)	143 microadenomas and 2 polyps
GD87	22	FAP	no	rectal polyps, desmoids
GD92	58	AFAP	n.a.	
GD94	65	AFAP	yes (vertical)	5 polyps, colon cancer
GD102	n.a.	Multiple polyposis	n.a.	cystadenocarcinoma (unknown site)
GD106	50	Multiple polyposis	n.a.	
GD107	42	Multiple polyposis	yes (horizontal)	
GD109	52	Multiple polyposis	no	36 polyps, colon cancer
GD112#1	37	FAP	yes (vertical)	colon, gastric, duodenal and rectal polyps
GD112#2	14	FAP	yes (vertical)	colon and gastric polyps
GD117	63	Multiple polyposis	yes (horizontal)	10 polyps, gastric cancer
GD118	71	AFAP	yes (vertical)	10 polyps, colon cancer
GD121	41	AFAP	n.a.	
GD122	59	Colon cancer	yes (vertical)	
GD123	60	AFAP	no	colon cancer
GD140	37	AFAP	no	gastric polyps
GD146	44	AFAP	no	colon and breast cancer
GD153	47	AFAP	yes (horizontal)	hyperplastic polyps, colon cancer
GD154	44	FAP	no	836 polyps
GD157	53	FAP	no	colon cancer
GD158	52	FAP	yes (vertical)	
GD159	26	FAP	yes (vertical)	rectal cancer
GD160	52	AFAP	no	10 polyps

### Analyses of *APC* and *MUTYH* mRNA expression

Patients analyzed by qRT-PCR belonged to three different subgroups: carriers of *APC* mutations (*APC*+, n. 13), carriers of *MUTYH* mutations (*MUTYH*+, n. 5) and patients negative at mutation screening for both genes (*APC*-/*MUTYH*-, n. 21). 2^−^∆Ct mean values of cases were compared to those of 42 healthy blood donors selected according similar gender and age in order to have a comparable sample size. RNA amounts from ten patients were used up for qRT-PCR analysis.

**Fig. 1:**
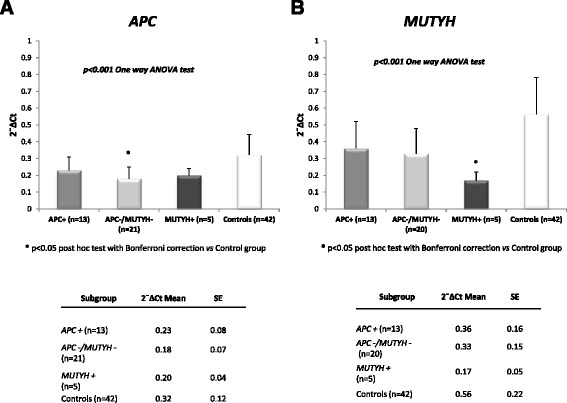
Germline expression by qRT-PCR of the *APC* and *MUTYH* genes. The figure shows the results from qRT-PCR analysis conducted in 39 patients carrying either *APC* mutation, *MUTYH* mutation or none mutations in both genes, compared to 42 controls. mRNA isolated from PBMCs was quantified by qRT-PCR using *GUSB* gene as endogenous control. Data were analyzed using the comparative Ct method and are expressed as mean and standard error (SE) of relative expression, which corresponds to the 2^−^ΔCt value; *P < 0.05 versus control group with post hoc test. **a** Relative expression levels of *APC* gene and associated table showing 2^−^∆Ct mean values ± SE of each subgroup. **b** Relative expression levels of *MUTYH* gene and associated table showing 2^−^∆Ct mean values ± SE of each subgroup

Analysis of *APC* gene expression by qRT-PCR showed a statistically significant reduced expression in the 21 *APC*-/*MUTYH*- patients (mean value of 0.18 ± 0.07 2^−^∆Ct) as compared to the control group (mean value of 0.32 ± 0.12 2^−^∆Ct) (*P* < 0.05) (Fig. 1a). On the other hand, *APC* expression in the 13 *APC*+ and 5 *MUTYH*+ patients, although lower compared to control individuals, did not show statistical significance (Fig. 1a).

Regarding *MUTYH* gene expression, qRT-PCR analysis showed a statistically significant reduced expression in the 5 *MUTYH*+ patients (mean value of 0.17 ± 0.05 2^−^∆Ct) as compared to the control group (mean value of 0.56 ± 0.22 2^−^∆Ct) (*P* < 0.05), while the 13 *APC*+ and the 20 *APC*-/*MUTYH*- patients did not show significant *MUTYH* reduction (Fig. 1b). One case, GD117, resulted not informative by *MUTYH* qRT-PCR (Additional file 1: Table S1).

Altogether, these data suggest that reduced *APC* expression may be detected even in the absence of *APC* germline mutation and that *MUTYH* mutations may be associated with reduced *MUTYH* expression.

### ASE analysis of *APC* and *MUTYH* genes

Germline ASE of *APC* was performed in 20 (out of 49) cases heterozygous for the SNP rs2229992 (c.1458C > T), of which 7 carrying *APC* mutation (Additional file 1: Table S1). To investigate if variations in ASE values can contribute to forms of colorectal disease with different degree of penetrance, the values were compared to data obtained from 53 consecutive CRC patients previously analyzed by the same assay [11]. We included one CRC case (case 19) turned out to be a *de novo* FAP in previous study, among *APC* mutation positive cases. A total of 21 polyposis and 52 consecutive CRC patients, were compared to 68 previously analyzed control individuals [11]. Results of ASE analysis showed in one case, GD41, bearing the p.Ser895X mutation, a germline allelic loss. The mean and median values are reported in Table 3, excluding the case GD41 without a quantifiable ASE value. The distribution of ASE values was tested for normality (Shapiro-Wilk normality test) and both *APC* mutation-positive and -negative FAP cases were distributed normally (respectively *P* = 0.499 and 0.682), whereas CRC cases and controls were not (Table 3). Only *APC* mutation-negative FAP cases had median and mean ASE values slightly, but significantly lower than controls by Kruskal-Wallis test (Table 3). Four out of 8 *APC* mutation-positive cases showed an imbalanced ASE, with values more than 1 SD from the overall mean (<0.99 and >1.53) compared to controls (Table 4; *P* = 0.022). Furthermore the group of the CRC cases deviating more than 1 SD from the overall mean was significantly larger than controls (Table 4; *P* = 0.001), as noted before [11]. Altogether, as regards APC mRNA expression, four patients with imbalanced ASE (GD22, GD41, GD72, GD82#1) showed also low levels of APC expression by qRT-PCR (Additional file 1: Table S1).

**Table 3:** Mean and Median ASE values of cases and controls for *APC* c.1458C > T assay

Status	N	Mean (±SD)	Median	Min	Max	Shapiro-Wilk	Multiple comparison of Kruskall-Wallis vs control
Controls^b^	68	1.25 (0.21)	1.20	0.89	1.92	**0.014**	
*APC* + polyposis cases	7^a^	1.20 (0.41)	1.38	0.43	1.60	0.499	0.560
*APC* - polyposis cases	13	1.11 (0.18)	1.11	0.83	1.51	0.682	**0.029**
CRC cases^b^	52	1.30 (0.32)	1.28	0.68	2.46	**0.041**	0.350
Overall	140	1.26 (0.27)	1.22	0.43	2.46		

^a^Case GD41 was not considered in the table because it showed monoallelic expression

^b^Previously published [11]

Bold values indicate statistical significance

**Table 4:** Distribution of cases and controls at 1.0 SD from the overall mean ASE for *APC* c.1458C > T assay

More than 1.0 SD from the overall mean (<0.99 and >1.53)				
Status	Within 1 SD	Out of 1 SD	Total	Chi-square or Fisher’s Exact *p*-value vs control
Controls^b^	57	11	68	
*APC* + polyposis cases	4	4^a^	8	**0.022**
*APC* - polyposis cases	9	4	13	0.214
CRC cases^b^	30	22	52	**0.001**
Overall	100	41	141	

^a^Case GD41, with monoallelic expression, was included in the table among cases deviating more than 1.0 SD from the overall mean

^b^Previously published [11]

Bold values indicate statistical significance

As regards *MUTYH*, all *APC* negative cases, heterozygous at *MUTYH* c.1014G > C variant, and cases bearing the c.536A > G (Y179C) pathogenic variant, were analyzed for ASE using 2 *primer extension* assays developed in this study. The ASE assay for *MUTYH* c.1014G > C variant was performed in 14 out of 49 cases (Additional file 1: Table S1) and 34 control individuals resulted heterozygous for the variant. The ASE assay for *MUTYH* c.536A > G (Y179C) pathogenic variant was performed in 5 cases (Additional file 1: Table S1). One case (GD68) has been analysed for both *MUTYH* assays. Considering that the means of ASE assays using the c.1014G > C and the c.536A > G variants were very similar (CV 4.28 % of the two means) we calculated the overall mean of all ASE values measured in cases and controls with both assays and that was 0.96 (SD ± 0.14; CV 14.51 %); the range of variation ± 1 SD was <0.82 and >1.10 (Additional file 2: Tables S2 and Additional file 3: Table S3). There were slight differences in mean and median ASE values in cases and controls. None of the individuals showed monoallelic expression or marked ASE. Results from *MUTYH* ASE analysis showed modest imbalances of values deviating more than 1 SD from the mean, in several individuals among cases and controls, but the differences were not statistical (Additional file 3: Table S3).

**Fig. 2:**
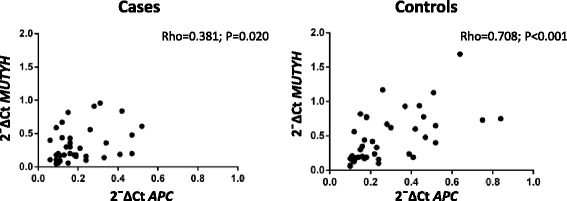
Correlation between *APC* and *MUTYH* gene expression. The figure shows *APC* and *MUTYH* absolute mRNA expression values measured in 39 cases (*left*) and 42 controls (right) correlated by Spearman’s test: correlation coefficient (rho) and *P* values (p)

We also correlated the *APC* and *MUTYH* gene expression in 39 cases and 42 controls by Spearman’s test. As reported in Fig. 2, the two genes were positively correlated both in cases and in controls (respectively Rho = 0.381, *P* = 0.020 e Rho = 0.708, *P* < 0.001). This correlation was more strengthened among controls.

## Discussion

In this study we analyzed mutational status, transcript dosage and disease phenotype, to investigate the role of altered gene expression in the pathogenesis of familial colorectal tumors. The results of this study showed that at germline level gene expression of both *APC* and *MUTYH* was reduced in hereditary colorectal polyposis patients compared to healthy donors. In particular a reduced *APC* expression was observed even in patients without *APC* germline mutation meanwhile a reduced *MUTYH* expression has been detected in patients with *MUTYH* mutations. The statistically significant reduction in *APC* expression detected by qRT-PCR in 21 cases with *APC*-/*MUTYH*- genotype (Fig. 1a) is in agreement with previous studies [16, 28]. As far as ASE of *APC,* marked imbalances (>2fold) were detected only in two *APC*+ patients included in this study. Other patients had only modest degrees of imbalances of *APC,* which, as previously discussed, increase the risk of colorectal cancer [11]. Among the 14 cases that could be analyzed by both qRT-PCR and ASE, 6 showed no altered expression by both methods, 4 (GD22, GD41, GD72, GD82#1) showed reduced expression and altered ASE, 2 showed (GD68, GD84) reduced expression by qRT-PCR with a balanced allelic expression, likely related to decreased expression of both alleles, and 2 had only modest degrees of imbalanced expression which might reflect a small reduction in the expression of one allele compensated by a small increase in the expression of the other (GD33, GD123) (Additional file 1: Table S1, Additional file 3: Table S3). A noteworthy observation comes from the allelic expression analysis of case GD41, carrier of the p.Ser895X *APC* mutation. In this case ASE identified the complete loss of an allele that could not be seen with the simple observation by qRT-PCR even if this case, with monoallelic expression, showed an approximately 50 % reduction in the overall expression of *APC* (Additional file 1: Table S1). Among patients with *MUTYH+* genotype two cases, GD82#1 and GD82#2, belonging to the same family, compound heterozygotes and carriers of the same mutations, showed different levels of *APC* expression (Table 1, Additional file 1: Table S1). GD82#1, affected by multiple polyposis and ciecum carcinoma, showed a lower level of *APC* expression; for GD82#2, presenting colorectal carcinoma without polyposis, the expression level for *APC* gene was higher. It can be assumed that the difference in *APC* expression in the two affected brothers might be associated with their different phenotype.

The cause of mRNA reduced expression is not known and could be related to mutations in regions of the gene not analyzed by mutational screening. Because *APC* is an oncosuppressor with a dominant pattern of hereditary transmission, its decreased expression is expected to contribute to FAP predisposition in these mutation negative cases. Thus decreased expression of *APC* in mutation negative cases appears to be a promising indicator of FAP predisposition.

Regarding *MUTYH,* five out of six patients with mutations, analyzed by qRT-PCR, showed a statistically reduced expression of the *MUTYH* gene and four out of five were carriers of *MUTYH* Y179C mutation. Patients belonging to this group manifested an attenuated phenotype (with absence of family history or horizontal transmission), except case GD72, who developed a classical FAP with vertical transmission (Table 1); these findings are in line with several studies [9, 27, 29].

Case GD72, carrier of a monoallelic Y179C mutation and showing by qRT-PCR a reduced expression (0.06 ± 0.01, Additional file 1: Table S1), has been analyzed by ASE of *MUTYH* and *APC* genes. In *MUTYH* the two alleles showed a quantitatively similar expression (1.08); as regarding *APC,* we found a moderately reduced relative expression (0.83) (Additional file 1: Table S1). In this patient both *MUTYH* alleles showed a low but equal expression with a reduction in absolute mRNA expression. This might explain FAP phenotype in a carrier of monoallelic *MUTYH* mutation. It is noteworthy that case GD72 manifested the disease at the age of 29 years. Individuals carrying monoallelic mutations tend to have a later presentation of disease of at least 5 years compared to biallelic ones but patients with heterozygous Y179C mutation have usually a more severe phenotype with earlier onset as compared with the other frequent G396D mutation [9, 30]. The missense Y179C mutation, situated in the catalytic domain of *MUTYH*, led to the formation of an inactive protein, as it can remove normal interactions with other proteins [31] and causes a quantitative reduction of mRNA, which would lead to reduced protein levels. Further studies on protein in those cases with both mutations and reduced expression could support our data on MUTYH protein functionality.

The risk of colorectal cancer in heterozygous carriers of *MUTYH* mutations has been considered comparable with that of first-degree relatives of patients with sporadic colorectal cancer by some authors [32] or considered increased due to a synergism with alterations in other genes involved in BER or other reparative pathways [9, 33]. Other authors assume that a slightly increased risk of cancer related to the presence of a monoallelic inactivation of *MUTYH* could be attributed to a situation of haploinsufficiency [34]. A complete picture of the effect of monoallelic mutations on the variability of clinical phenotype linked to *MUTYH* is still to be defined. Studies of absolute and relative gene expression applied to a larger group of monoallelic mutation carriers could provide a clue of possible involved pathways.

Correlation between germline altered gene expression and disease, sometimes in absence of mutations, have been reported in chronic inflammatory diseases and in forms of intellectual disability, all compared to control individuals [35–37]. In spite of several evidences that showed as different phenotypes can be classified based on degree of severity of polyposis and site of *APC* gene mutation [8, 21, 27, 38, 39], there is a scarcity of data concerning correlations between disease manifestations and *APC* germline reduced expression. The observations in relatives GD82#1 and GD82#2 might be the first evidence of this kind of correlations. It will be very interesting to extend the study to other polyposis families with a larger number of relatives. If confirmed in further studies, the correlation between germline reduced expression and disease manifestations could improve the predictive genetic diagnosis of at-risk individuals belonging to families with reduced mRNA expression regardless of presence of mutation.

Among the various cellular functions of *APC* gene, it was recently discovered its involvement in DNA repair mediated by BER, as it is able to modulate the activity of this pathway [40]. Therefore we searched for possible mutual modulation between *APC* and *MUTYH* expression in 39 cases and 42 controls by Spearman’s test. As reported in Fig. 2, the two genes were positively correlated both in cases and in controls although the significance seemed to be stronger among controls. A modulation between APC and MUTYH was also observed in Fig. 1: carriers of mutation compared to cases without mutations in one gene showed a higher expression of the other gene.

## Conclusions

In this study the most significant result comes from the analysis of *APC* and *MUTYH* expression levels in hereditary colorectal polyposis patients carrying either *APC* mutation, *MUTYH* mutation or none mutations in both genes, compared to 42 controls. Both genes showed a reduced germline expression, not always corresponding to gene mutation. Expression of *APC* is decreased in mutation negative cases and this appears to be a promising indicator of FAP predisposition, while for *MUTYH* gene, reduced mRNA expression is associated to mutation. This is the first evidence of ASE analysis of *MUTYH* but, as the recessive pattern of hereditary transmission of this gene, altered ASE could be masked by contemporary reduced expression of both alleles. This study could improve the predictive genetic diagnosis of at-risk individuals belonging to families with reduced mRNA expression regardless of presence of mutation. It will be very interesting to extend the study to other genes of the Wnt and BER pathways or to other known cancer predisposing genes.

## Additional files

Additional file 1: Table S1. qRT-PCR and ASE results from 50 cases investigated. (DOCX 37 kb) Additional file 2: Table S2. Mean and median ASE values of cases and controls after combining both *MUTYH* assays. (DOCX 18 kb) Additional file 3: Table S3. Distribution of cases and controls at 1 SD from the overall mean ASE after combining both *MUTYH* assays. (DOCX 41 kb)

## Abbreviations

CRC: Colorectal cancer
ASE: Allele-specific expression
APC: Adenomatous polyposis coli
MUTYH: Mut Y homolog
PBMCs: Peripheral blood mononuclear cells
qRT-PCR: Quantitative real-time PCR
FAP: Familial adenomatous polyposis
MAP: *MUTYH*-associated polyposis
BER: Base excision repair
ROS: Reactive oxygen species
cDNA: Complementary DNA
dHPLC: Denaturing high performance liquid chromatography
MLPA: Multiplex ligation-dependent probe amplification
mRNA: Messenger RNA
GUSB: B-Glucuronidase
SNP: Single nucleotide polymorphism
SD: Standard deviation
MAF: Minor allele frequency
gDNA: Genomic DNA
