# Supplementary material for: Correlation between mutations and mRNA expression of APC and MUTYH genes: new insight into hereditary colorectal polyposis predisposition
Source: J Exp Clin Cancer Res. 2015 Oct 28;34:131. doi: 10.1186/s13046-015-0244-4 (PMC4625907; doi:10.1186/s13046-015-0244-4)
Supplement: Additional file 1: Table S1. — qRT-PCR and ASE results from 50 cases investigated. (DOCX 37 kb) [file 13046_2015_244_MOESM1_ESM.docx]

**Additional table 1.** qRT-PCR and ASE results from 50 cases investigated.

| qRT-PCR (2¯∆Ct) | | | | | | ASE value^a^ | | | |
| --- | --- | --- | --- | --- | --- | --- | --- | --- | --- |
| Sample | ***APC*** | **SE** | ***MUTYH*** | **SE** | ***APC***  **c.1458C>T** | | **CV (%)** | ***MUTYH* c.1014G>C; c.536A>G** | **CV (%)** |
| GD22^b^ | 0.09 | 0.01 | 0.11 | 0.01 | **1.54** | | 5.58 |  |  |
| GD23^b^ | n.a. | n.a. | n.a. | n.a. | 1.03 | | 14.97 |  |  |
| GD31^b^ | 0.11 | 0.01 | 0.09 | 0.01 |  | |  |  |  |
| GD33^b^ | 0.28 | 0.02 | 0.91 | 0.04 | **1.60** | | 8.42 |  |  |
| GD37^b^ | 0.47 | 0.01 | 0.20 | 0.02 | 1.39 | | 13.83 |  |  |
| GD38^b^ | 0.34 | 0.02 | 0.36 | 0.03 | 1.02 | | 2.03 |  |  |
| GD41^b^ | 0.16 | 0.01 | 0.36 | 0.03 | **m.a.** | |  |  |  |
| GD48^b^ | 0.26 | 0.02 | 0.56 | 0.06 | 1.38 | | 10.34 |  |  |
| GD57^b^ | 0.06 | 0.01 | 0.11 | 0.01 |  | |  |  |  |
| GD58^b^ | 0.16 | 0.01 | 0.43 | 0.01 |  | |  |  |  |
| GD59^b^ | 0.42 | 0.01 | 0.84 | 0.04 |  | |  |  |  |
| GD68^b^ | 0.14 | 0.01 | 0.30 | 0.02 | 1.11 | | 14.41 | 1.04  0.90^c^ | 9.17  10.09^c^ |
| GD70 | 0.06 | 0.01 | 0.40 | 0.02 |  | |  | 1.03 | 4.12 |
| GD72^b^ | 0.15 | 0.01 | 0.06 | 0.01 | **0.83** | | 14.96 | 1.08^c^ | 14.20^c^ |
| GD74^b^ | 0.12 | 0.01 | 0.44 | 0.01 |  | |  |  |  |
| GD78 | 0.28 | 0.02 | 0.67 | 0.06 |  | |  |  |  |
| GD80 | n.a. | n.a. | n.a. | n.a. | **0.97** | | 3.25 |  |  |
| GD81 | 0.52 | 0.05 | 0.61 | 0.04 | 1.23 | | 7.08 |  |  |
| GD82#1^b^ | 0.18 | 0.02 | 0.18 | 0.01 | **0.94** | | 15.58 | 1.06^c^ | 1.00^c^ |
| GD82#2^b^ | 0.33 | 0.02 | 0.14 | 0.00 |  | |  | 1.01^c^ | 16.18^c^ |
| GD83 | n.a. | n.a. | n.a. | n.a. | 1.19 | | 7.37 | **1.17** | 15.75 |
| GD84 | 0.11 | 0.01 | 0.17 | 0.02 | 1.51 | | 7.27 |  |  |
| GD86 | 0.21 | 0.01 | 0.28 | 0.01 | 1.23 | | 3.70 |  |  |
| GD87 | 0.16 | 0.07 | 0.30 | 0.09 |  | |  |  |  |
| GD91^b^ | 0.19 | 0.04 | 0.16 | 0.01 |  | |  |  |  |
| GD92 | 0.31 | 0.04 | 0.96 | 0.06 |  | |  |  |  |
| GD94 | 0.09 | 0.02 | 0.18 | 0.01 |  | |  |  |  |
| GD102 | 0.09 | 0.01 | 0.59 | 0.05 |  | |  | 0.90 | 9.38 |
| GD103^b^ | 0.09 | 0.01 | 0.05 | 0.00 |  | |  |  |  |
| GD106 | 0.10 | 0.01 | 0.20 | 0.02 |  | |  |  |  |
| GD107 | 0.19 | 0.02 | 0.18 | 0.02 |  | |  |  |  |
| GD109 | 0.10 | 0.01 | 0.06 | 0.00 |  | |  | 0.94 | 6.57 |
| GD112#1 | 0.16 | 0.00 | 0.20 | 0.02 |  | |  |  |  |
| GD112#2 | 0.13 | 0.01 | 0.18 | 0.01 |  | |  |  |  |
| GD117 | 0.04 | 0.00 | n.a. | n.a. |  | |  | 0.98 | 11.36 |
| GD118 | 0.06 | 0.00 | 0.05 | 0.00 |  | |  |  |  |
| GD119^b^ | 0.41 | 0.02 | 0.19 | 0.01 |  | |  |  |  |
| GD121 | 0.24 | 0.02 | 0.10 | 0.00 | 1.17 | | 11.36 |  |  |
| GD122 | 0.10 | 0.01 | 0.07 | 0.00 |  | |  | **1.12** | 4.87 |
| GD123 | 0.47 | 0.03 | 0.48 | 0.05 | **0.97** | | 4.03 |  |  |
| GD140 | 0.24 | 0.07 | 0.16 | 0.01 |  | |  |  |  |
| GD146 | 0.15 | 0.01 | 0.82 | 0.10 |  | |  | 0.93 | 7.85 |
| GD153 | n.a. | n.a. | n.a. | n.a. | 1.00 | | 11.18 | 0.86 | 9.40 |
| GD154 | n.a. | n.a. | n.a. | n.a. | 1.22 | | 16.75 | **0.79** | 4.43 |
| GD155^b^ | n.a. | n.a. | n.a. | n.a. | 1.11 | | 16.46 | 1.05^c^ | 3.38^c^ |
| F100 | n.a. | n.a. | n.a. | n.a. |  | |  | 1.09 | 10.91 |
| F137 | n.a. | n.a. | n.a. | n.a. |  | |  | 0.88 | 3.21 |
| AF186 | n.a. | n.a. | n.a. | n.a. |  | |  | **0.80** | 18.69 |
| AF200 | n.a. | n.a. | n.a. | n.a. |  | |  | 0.93 | 15.38 |
| Case 19 | n.a. | n.a. | n.a. | n.a. | **0.43** | | 2.69 |  |  |

n.a.= not available; m.a.= monoallelic

^a^analysis performed on heterozygous cases (see Material and Methods)

^b^patients carrier of *APC* and *MUTYH* mutations

^c^results from *MUTYH* c.536A>G assay

ASE values outside 1.0 SD are in bold
