# Supplementary material for: Correlation between mutations and mRNA expression of APC and MUTYH genes: new insight into hereditary colorectal polyposis predisposition
Source: J Exp Clin Cancer Res. 2015 Oct 28;34:131. doi: 10.1186/s13046-015-0244-4 (PMC4625907; doi:10.1186/s13046-015-0244-4)
Supplement: Additional file 2: Table S2. — Mean and median ASE values of cases and controls after combining both MUTYH assays. (DOCX 18 kb) [file 13046_2015_244_MOESM2_ESM.docx]

**Additional Table 2.** Mean and median ASE values of cases and controls after combining both *MUTYH* assays

|  | **N** | **Mean (±SD)** | **Median** | **Min** | **Max** | **Shapiro-Wilk normality** | **Student t test** |
| --- | --- | --- | --- | --- | --- | --- | --- |
| **Controls** | 34 | 0.96 (0.16) | 0.94 | 0.66 | 1.23 | <0.001 | 0.638 |
| ***APC* - cases** | 18 | 0.98 (0.11) | 0.98 | 0.79 | 1.17 | 0.672 |  |
| **Total** | 52 | 0.96 (0.14) | 0.95 | 0.66 | 1.23 |  |  |
