# Supplementary material for: Correlation between mutations and mRNA expression of APC and MUTYH genes: new insight into hereditary colorectal polyposis predisposition
Source: J Exp Clin Cancer Res. 2015 Oct 28;34:131. doi: 10.1186/s13046-015-0244-4 (PMC4625907; doi:10.1186/s13046-015-0244-4)
Supplement: Additional file 3: Table S3. — Distribution of cases and controls at 1 SD from the overall mean ASE after combining both MUTYH assays. (DOCX 41 kb) [file 13046_2015_244_MOESM3_ESM.docx]

**Additional Table 3.** Distribution of cases and controls at 1 SD from the overall mean

ASE after combining both *MUTYH* assays

| **More than 1.0 SD from the overall mean (<0.82 and >1.10)** | | | | |
| --- | --- | --- | --- | --- |
| **Status** | **Within 1 SD** | **Out of 1 SD** | **Total** | **Fisher's Exact p-value** |
| **Controls** | 18 | 16 | 34 | 0.134 |
| ***APC* - cases** | 14 | 4 | 18 |  |
| **Total** | 32 | 20 | 52 |  |
